# Assessment of Gliflozins prescribing pattern in a United Arab Emirates tertiary-level care hospital

**DOI:** 10.3389/fphar.2025.1529528

**Published:** 2025-04-01

**Authors:** Wessa Shenouda, Dixon Thomas, Omar Nabi, Seeba Zachariah

**Affiliations:** ^1^ College of Pharmacy, Gulf Medical University, Ajman, United Arab Emirates; ^2^ Operations, Thumbay University Hospital, Ajman, United Arab Emirates

**Keywords:** Gliflozins, SGLT2 inhibitors, drug utilization, diabetes mellitus, heart failure, chronic kidney disease, UAE

## Abstract

**Background:**

Sodium-Glucose Co-Transporter 2 (SGLT2) inhibitors, known as Gliflozins, have demonstrated efficacy in managing type 2 diabetes mellitus (T2DM) and providing cardiovascular and renal benefits. Given the prevalence of diabetes, heart failure (HF), and chronic kidney disease (CKD) in the UAE, there is a need to evaluate the prescribing patterns of Gliflozins in these population. The objective of this study was to explore the relationship between Gliflozins use for patients who were admitted to the hospital at least once from 2021 to 2023 and different clinical factors.

**Methods:**

A retrospective medication review was conducted from 2021 to 2023 at tertiary-level care hospital in Ajman, UAE. Data were collected on prescribed Gliflozins, patient demographic information, BMI, HbA1c levels, and comorbidities (HF, CKD). Chi-square tests and binary logistic regression were used to explore associations between Gliflozin use and clinical factors.

**Results:**

Out of the 255 patients’ data collected, Gliflozin use was significantly associated with obesity (p = 0.002), higher HbA1c levels (p < 0.001), and comorbidities, particularly HF (61.5% of HF patients) and CKD. The use of Gliflozins increased each year. Patients with HF were 8.03 times more likely to use Gliflozins, and those with diabetes were 6.86 times more likely, underscoring the multidimensional role of these medications.

**Conclusion:**

Gliflozin prescribing patterns in the UAE reflect global trends, with increased use among patients with diabetes, HF, and CKD. Further research is recommended to explore factors influencing prescription practices and optimize Gliflozin therapy if gliflozins use considerably increase in new diagnosis of diabetes and CKD even in mild conditions.

## Introduction

The main goals of managing the diabetes are to achieve optimal glycemic control and to stop the development and progression of microvascular and macrovascular complications. However, treatment modalities vary based on the individual needs of each patient. Gliflozins also known as Sodium Glucose Co-Transporter 2 (SGLT2) inhibitors were mainly introduced to treat (T2DM) type 2 diabetes mellitus as an add-on therapy (ADA, 2022; Prosperi et al., 2023; Verdecchia et al., 2023; Yaribeygi et al., 2023).

SGLT2 inhibitors or Gliflozins mainly block the SGLT2 transporter in the proximal renal tubules of the kidneys. This reduces the reabsorption of filtered glucose while increasing urine glucose excretion. These medications act by specifically inhibiting the SGLT2 co-transporters in the proximal renal tubule, preventing filtered glucose and sodium from being reabsorbed. This method of action is independent of insulin sensitivity; Gliflozins are a unique treatment approach that directly targets the kidneys (Abdelgani et al., 2023; Madonna, 2021; Bragagni et al., 2021). SGLT2i consist of five oral drugs: Canagliflozin, dapagliflozin, empagliflozin, ertugliflozin, and sotagliflozin (Helvacı and Helvacı, 2023; Haas et al., 2014). These medications have been found to provide considerable metabolic, renal, and cardiovascular benefits, including lower levels of glycated hemoglobin (HbA1c), blood pressure, and body weight (Varshney and Rawat, 2023; Begić and Aziri, 2023; Delanaye and Scheen, 2023; Zelniker et al., 2019; Perkovic et al., 2019). The cardiovascular benefits of empagliflozin, dapagliflozin, and other SGLT2i, respectively, in lowering the risk of HF hospitalization and cardiovascular diseases (CVD) mortality among patients with type 2 diabetes have been well proven in clinical trials EMPA-REG OUTCOME, DECLARE-TIMI 58, and DAPA-HF (Tsigkou et al., 2023; Elendu et al., 2023; Rahhal et al., 2023; Arbel et al., 2021; McGuire et al., 2020; Täger et al., 2020; Thiagaraj et al., 2023; Kluger et al., 2018).

The clinical trials, including the CREDENCE and DAPA-CKD trials, have demonstrated the renoprotective effects of SGLT2i in patients with CKD, irrespective of their diabetic status (Heerspink et al., 2020; Jongs et al., 2021). These trials revealed significant reductions in the risk of kidney disease, progression of albuminuria, and cardiovascular events among CKD patients receiving SGLT2i therapy. Additionally, SGLT2i have been shown to improve glomerular hemodynamics, reduce renal inflammation and fibrosis, and mitigate oxidative stress, all of which contribute to their renoprotective effects (Skrabic et al., 2022). Analysis where patients diagnosed with HF showed more prescriptions of Gliflozin as compared to CKD patients (p < 0.001). The heart failure has emerged as one of the leading indications for Gliflozin therapy in non-diabetic patients as well. Both the DAPA-HF and EMPEROR-Reduced trials showed that among patients with reduced ejection fraction (including those without diabetes), some benefit from Gliflozins in reducing the risk of cardiovascular death/heart failure hospitalization (McMurray et al., 2019; Packer et al., 2020; Zannad et al., 2020; McGuire et al., 2021; Padda et al., 2023).

Gliflozins, should not be used in patients with the following cases: type 1 diabetes (T1DM), volume insufficiency, injection of contrast medium, ketoacidosis, hypersensitivity to Gliflozins, or severe renal impairment (Pistelli et al., 2023). Caution to be exercised when prescribing SGLT2 inhibitors alongside insulin or insulin secretagogues, as this combination may increase the risk of hypoglycemia (Faillie, 2017). Many studies have demonstrated that Gliflozins not only lower HbA1c levels but also offer extra benefits for heart and kidney health (Savarese et al., 2021; Zinman et al., 2015; Neal et al., 2017; Wiviott et al., 2019). Safety and effectiveness of SGLT2i in patients with heart failure continue to evolve with positive safety profiles, as well as decreases in heart failure hospitalizations and cardiovascular mortality, hence bolstering the drug’s use in HF treatment (Nassif et al., 2019; Benvenuto et al., 2023; Hinton et al., 2021; Nagasu et al., 2021; Butler et al., 2020).

Despite the growing body of evidence supporting the cardiovascular and renal benefits of SGLT2i, there remains a notable gap in our understanding of their prescribing patterns and real-world effectiveness in tertiary care hospitals in the United Arab Emirates (UAE). Given the unique demographic and clinical characteristics of the UAE population, including a high prevalence of DM and its associated comorbidities, there is a compelling need to evaluate the utilization of SGLT2i in this context. By elucidating the current prescribing practices, adherence to guidelines, and clinical outcomes associated with SGLT2i therapy in patients with HF and CKD at a tertiary care hospital in the UAE, this study aims to address existing knowledge gaps and inform evidence-based decision-making in clinical practice. The objective of this study was to explore the relationship between Gliflozins use for patients who were admitted to the hospital at least once from 2021 to 2023 and different clinical factors.

## Materials and methods

### Research design

This study used a retrospective medication records review, covering the period from January 2021 to December 2023. The study focused on patients who had been prescribed Gliflozins during this timeframe at a tertiary care hospital in the UAE, adhering to specific inclusion and exclusion criteria.

### Study site

The study was conducted at a tertiary care facility located in the Emirate of Ajman in the United Arab Emirates (UAE). The Hospital is one of the largest private academic hospital in the Middle East region, with a capacity of 350 beds. Patients from Ajman and nearby Emirates are being treated or followed-up for their conditions at the study site. The hospital has internal medicine, cardiac, and nephrology clinics.

### Study population

The data of patients with diagnosis of diabetes mellitus, HF, and CKD were collected whether they were prescribed with gliflozins or not. The medical records reviewed was of in-patients thus the patients who were not admitted to the hospital at least once during the study period were not reviewed. Only the patients who were admitted to the hospital were studied initially as adoption of Gliflozins is more likely in those who are admitted, not in mild out-patient cases. Data of patients diagnosed with type 1 diabetes mellitus were excluded. Data of patients with largely missing data was also excluded.

### Population size

As this was a population-based study, no sample size calculation was done. All eligible patients at the Hospital in the timeframe were included in the study as per the criteria. Data collection was conducted retrospectively, ensuring that patients meeting the specified inclusion and exclusion criteria were comprehensively captured.

### Participants criteria

Participants were selected according to the following criteria.

#### Inclusion criteria

The study included patients with CVDs such as post-myocardial infarction (MI), HF, ischemic heart disease (IHD), atrial fibrillation (AF), hypertension (HTN), stroke, and/or DM, Additionally, patients with CKD were included. All patients whether they were prescribed of Gliflozins or not.

#### Exclusion criteria

Data of patients diagnosed with type 1 DM, data of patients with largely missing data was also excluded, and hypersensitivity patients to Gliflozins were excluded from the study.

### Ethics approval

Prior to commencement, ethical approval for this study was obtained from the Institutional Review Board (IRB) approval at the study site. The approval was granted on June 06, 2023, Ref No. IRB-COP-STD-108-JUNE-2023. Patient confidentiality has been maintained.

### Data collection

A data collection form was developed in line with the study objectives and was used to gather relevant data looking at similar studies. The collected data included patient profile details such as gender, age, BMI, number of patients from 2021 to 2023, diabetes comorbidities such as CKD and HF, diagnoses, lab test results (including the latest HbA1c reading), and medication prescription history. This information was entered into a Microsoft Excel file for further analysis using Excel and SPSS, ensuring a record of the raw data was maintained. The research team held regular meetings at least once per week to review, discuss, and validate the collected data and its analysis.

### Data analysis

The data collected from the Excel sheet was analyzed using the Statistical Package for Social Sciences (IBM SPSS Statistics, Version 26.0, IBM Corp., Armonk, NY, United States). Data were presented as proportions, means (±SD), or medians and ranges, as appropriate. The statistical significance level was set at an alpha value of 0.05. Descriptive analysis was conducted for patients’ demographics (e.g., gender, age, BMI) and clinical characteristics (e.g., A1C). To determine the association of Gliflozin use with demographic and clinical factors, a Chi-square test was employed to assess if there is a significant association between two categorical variables. Additionally, a Binary Logistic Regression model was performed to investigate factors influencing Gliflozin use.

## Results

### Patients demographic characteristics and clinical profile

Data of a total of 255 patients were reviewed. The Table 1 shows majority being male (71.4%) and 28.6% female. The average age of the participants was 60 (±13) years. The distribution of the study population by age group showed that 95 (37.3%) were aged 56–70 years, 77 (30.2%) were 41–55 years old, 66 (25.9%) were over 70 years, and only 17 patients (6.7%) were at the young age group, i.e., 27–40-year. The body mass index (BMI) data reveals that 99 (38.8%) of patients were classified as obese, 91 (35.7%) as overweight, 60 patients (23.5%) had a normal BMI, and only five patients (2.0%) were underweight. Table 1 also presents the clinical characteristics and number of patients data for the participants. Across the 3 years studied, the highest proportion of number of patients was in 2023 (45.9%), followed by 27.8% in 2022 and 26.3% in 2021. The majority of patients had diabetes (72.9%), followed by chronic kidney disease (CKD) in 74.5% and heart failure (HF) in 25.5% of the patient population. Notably, data shows that more than half of the patients, 137 (54.6%), have both CKD and diabetes, 46 (18.3%) have diabetes with HF, and 183 (72.9%) suffer from all three conditions (CKD, HF, and diabetes). Despite the high prevalence of these conditions, only 33.3% of the patients were prescribed Gliflozins during the study period.

**TABLE 1:** Patients demographic and clinical characteristics of the patient population (n = 255).

Characteristics		N (%)
Gender	Female	73 (28.6)
	Male	182 (71.4)
Age Group	27–40 years	17 (6.7)
	41–55 years	77 (30.2)
	56–70 years	95 (37.3)
	>70 years	66 (25.9)
Average age in years (±SD)	60 (±13)	
Body Mass Index (BMI)	Less than 18.5 (Underweight)	05 (2.0)
	18.5–24.9 (Normal)	60 (23.5)
	25.0–29.9 (Overweight)	91 (35.7)
	≥30.0 (Obese)	99 (38.8)
Year	Year 2021	67 (26.3)
	Year 2022	71 (27.8)
	Year 2023	117 (45.9)
HbA1C (latest reading)	Normal (<5.7)	33 (12.9)
	Prediabetes (5.7–6.4)	48 (18.8)
	Diabetes (≥6.5)	96 (37.6)
	N/A^a^	78 (30.5)
Diagnosis	Heart failure	65 (25.5)
	Chronic kidney disease	190 (74.5)
	Diabetes	183 (72.9)
Comorbidities	Diabetes with CKD	137 (54.6)
	Diabetes with HF	46 (18.3)
	Diabetes with CKD and HF	183 (72.9)
Gliflozin medication used during the study period	Yes	85 (33.3)
	No	170 (66.7)

^a^ Not available data.

### Gliflozin medications prescribed during the study period

Majority of the patients were prescribed Empagliflozin 10, 12.5, 25 mg 55 (64.70%), Dapagliflozin 5, 10 mg 18 (21.17%), Canagliflozin 100, 300 mg 11 (12.94%), Ertugliflozin 5, 15 mg 1 (1.17%). Table 2 provides a summary of descriptive statistics for the participants (e.g., age, BMI, and HbA1c levels). The mean age of participants was 60.2 years, with a standard deviation of 12.0 years, ranging from 27 to 87 years. The average BMI was 31.7.

**TABLE 2:** Descriptive statistics of age, BMI, and HbA1c for participants prescribed Gliflozin during the study period (n = 85).

Variable	Mean (±SD)	Median	Min.	Max.	N
Age (years)	60.2 (12.0)	61	27	87	85
Body mass index (BMI)	31.7 (7.7)	30.5	16.2	62.7	85
HbA1c (%)	7.6 (1.5)	7.4	5.1	11.8	66

### Association of Gliflozin use with clinical factors

While Table 3 demonstrates the key associations between Gliflozin medication use and various factors. Notably, Gliflozin use was significantly higher among overweight or obese participants (38.5%) compared to those with a normal BMI (16.7%, p = 0.002). Patients with diabetes (HbA1c ≥ 6.5) also had a higher Gliflozin use rate (51.0%) compared to those with normal HbA1c levels (13.0%, *P* < 0.001). Additionally, diabetic patients generally had significantly higher Gliflozin use (39.9%) compared to non-diabetic patients (14.7%, *P* < 0.001). The presence of comorbidities such as heart failure and chronic kidney disease further increased Gliflozin use, with significant associations observed (*P* < 0.001).

**TABLE 3:** Association of Gliflozin use with demographic and clinical factors.

Variable	Category	Gliflozin use (No) N (%)	Gliflozin use (Yes) N (%)	Total N (%)	*P*-value
Gender	Female	53 (72.6)	20 (27.4)	73 (100)	0.203
	Male	117 (64.3)	65 (35.7)	182 (100)	
Age groups (years)	27–40	13 (76.5)	4 (23.5)	17 (100)	0.089
	41–55	52 (67.5)	25 (32.5)	77 (100)	
	56–70	55 (57.9)	40 (42.1)	95 (100)	
	>70	50 (75.8)	16 (24.2)	66 (100)	
BMI groups	18.5–24.99 (Normal)	50 (83.3)	10 (16.7)	60 (100)	0.002^a^
	25–29.9 - ≥30 (Overweight - Obese)	120 (61.5)	75 (38.5)	195 (100)	
HbA1c Group	Less than <5.7 (normal)	26 (78.8)	7 (21.2)	33 (100)	<0.001^a^
	5.7–6.4 (Prediabetes)	38 (79.1)	10 (20.9)	48 (100)	
	≥6.5 (Diabetes)	47 (49.0)	49 (51.0)	96 (100)	
Diagnosed with diabetes	Yes	110 (60.1)	73 (39.9)	183 (100)	<0.001^a^
	No	58 (85.3)	10 (14.7)	68 (100)	
Comorbidity/complications	HF	25 (38.5)	40 (61.5)	65 (100)	<0.001^a^
	CKD	145 (76.3)	45 (23.7)	190 (100)	
	CKD with diabetes	97 (70.8)	40 (29.2)	137 (100)	<0.001^a^
	CKD with no diabetes	46 (92.0)	4 (8.0)	50 (100)	
	HF with diabetes	13 (28.3)	33 (71.7)	46 (100)	
	HF with no diabetes	12 (66.7)	6 (33.3)	18 (100)	

^a^ Significant value. HF, Heart Failure; CKD, chronic kidney disease.

### Relationship between Gliflozins use and hospital number of patients

**FIGURE 1:**
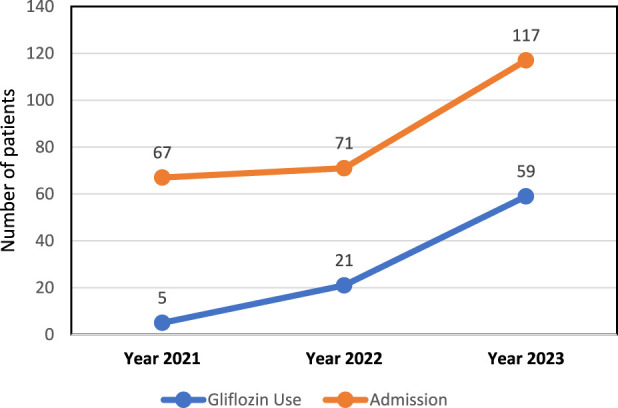
Pattern of Gliflozin Use and number of patients (2021–2023).

Data in Figure 1 shows a positive correlation between the increase in Gliflozin use and the rise in number of patients over the 3-year period. An increase in Gliflozin medication use and another significant increase in number of patients over the 3 years indicating that these two variables may be related.

### Factors influencing Gliflozin medication use

Data in Table 4 presents the results of a binary logistic regression analysis, highlighting significant factors associated with Gliflozins use. The adjusted odds ratios (AOR), along with their 95% confidence intervals (CI) and p-values, are provided. Key findings include a 3.57 times higher likelihood of Gliflozins use among participants with overweight and obese (BMI ≥25) compared to those with normal BMI (18.5–24.99). Though from 2021 to 2023 significant increase also has been shown, confidence intervals are large decreasing accuracy or prediction. The number of prescriptions also increased over this time with the highest odds observed in 2023 (AOR: 24.87). Individuals diagnosed with HF are 8.03 times more likely to use Gliflozins compared to those with CKD Additionally, diabetic patients were 6.86 times more likely to use Gliflozins than non-diabetics. The P-values indicate that all these factors are statistically significant predictors of Gliflozins use.

**TABLE 4:** Factors influencing Gliflozins, a binary logistic regression analysis.

Factor(s)	Adjusted odds ratio (AOR)	95% C.I.for AOR		P- value
		Lower	Upper	
BMI (Overweight - Obese groups)	3.573	1.507	8.472	0.004^a^
Year of prescription 2022	7.813	2.390	25.545	0.001^a^
Year of prescription 2023	24.870	7.919	78.108	0.001^a^
Diagnosis – Heart Failure	8.032	3.598	17.928	0.001^a^
Diabetic - Yes	6.855	2.803	16.761	0.001^a^

^a^ Significant value.

## Discussion

The demography of this study gives a glimpse of the population, which match with what is known from the literature. Known associated factors to diabetes are age and gender, BMI and others. Gender inequalities in health policy are most evident in the high prevalence of diabetes and CKD. The process of aging itself is a risk factor for the development of these diseases, and older adults, therefore, have a greater number of comorbidities. This was supported by the research of the Global Burden of Disease Study, which lays down the fact that as people get older, the risk of getting chronic diseases, more specifically, diabetes and CKD, is increasing incredibly as well (Shi et al., 2018; Chang et al., 2018; Vos et al., 2020). A large part of the study population is either overweight or obese (38.8% and 35.7%, respectively). Obesity is known to increase the risk of both diabetes and CKD. Studies have found that obesity makes the body less sensitive to insulin, causing diabetes, and also worsens kidney disease by increasing pressure in the kidneys and hyperfiltration (Wang et al., 2023). It is well established that lifestyle interventions, particularly in high-risk populations, can have a profound effect on preventing and managing chronic conditions (Jiang et al., 2022).

Diabetes, CKD and HF have all been identified as being at high risk of generating frequent number of patients, especially when presenting with less than optimal control or deteriorating to more advanced disease stages (Ponikowski et al., 2016; Zhou et al., 2018). In a similar terms, HF dramatically raises diabetes risk, and the development of HF requires special attention in the management of diabetes (Bozkurt et al., 2021). It was established that the major users of the SGLT2-inhibitors belong to middle-aged to elderly individuals due to the medications’ efficiency in managing diseases that are the result of diabetes (Huang et al., 2021).

One of the most noticeable results is the strong relationship between Gliflozin use and patients’ body mass index (BMI). Overweight and obese patients were much more likely to be given Gliflozin (38.5%) than those with a normal BMI (16.7%, p = 0.002). This matches with other research, as Gliflozins have been found to protect the heart and kidneys in obese people, who are more at risk for both diabetes and related problems (Ferrannini, 2017; Wanner et al., 2016; Brown et al., 2021; Jardine et al., 2020; Ang et al., 2022; Wada et al., 2022a; Wada et al., 2022b).

Another factor to consider is that the rise in hospital visits might be attributed to the early phases of Gliflozin medication. Some studies demonstrate that starting SGLT2 inhibitors may result in longer hospitalizations for complications such as diabetic ketoacidosis or low fluid levels, especially in elderly or frail patients (Zelnick et al., 2021; Fralick et al., 2017). While these early adverse effects were infrequent, they might have contributed to the increased hospitalization rates observed during the initial years of Gliflozin usage. It is also important to note that Gliflozin medication is frequently used in patients with advanced HF and CKD who have a high burden of hospitalizations. This is congruent with the findings of big real-world trials, such as the DELIVER study (Peikert et al., 2022; Scheen and Lancellotti, 2023). Furthermore, the COVID-19 pandemic overlapped part of the study period and may have affected hospitalization patterns. Gliflozins, though associated with some degree of beneficial effect on severe outcomes in people with diabetes hospitalized for COVID-19, may have been only a small contributor overall to the greater number of hospitalizations seen during the health crisis (Cai et al., 2021; Kosiborod et al., 2017).

Gliflozins can cause weight loss, improve insulin sensitivity, and reduce visceral fat. With such strong evidence, it makes sense that Gliflozins were often prescribed to heart failure patients in this study, highlighting their value in slowing the worsening of heart failure and improving patient health (Ferrannini and Solini, 2012; Tornyos et al., 2022). Furthermore, the preventive benefits of Gliflozins extend beyond glucose reduction, which has led to their wider usage in patients with diabetes who are at high risk for cardiovascular and renal consequences (Folkerts et al., 2021; Singh et al., 2024).

While this study provides valuable findings, several limitations must be acknowledged. The population size of 255 participants may limit the generalizability of the findings to a larger hypertensive and Gliflozins patient population. The study is conducted in a specific tertiary care hospital in the UAE, which may not be representative of other healthcare settings or populations, potentially limiting the external validity of the results. Data of patients at least admitted once to the hospital during the study period is excluding data of those who were not admitted. Approximately 30.5% of patients had no HbA1c recorded data available, which could have affected the accuracy of the results regarding the association between diabetes control and Gliflozin use.

## Conclusion

The binary logistic regression analysis shows that Gliflozins use increased at study site from 2021 to 2023. Gliflozin use was significantly associated with obesity, higher HbA1c levels, and comorbidities, particularly HF and CKD. Patients with diabetes or HF were more likely to be prescribed with Gliflozins. CKD and HF indications without diabetes shall increase in the future. Future studies shall expect to capture further growing trends of Gliflozins prescribing including those who are not being admitted to the hospital.

As more information supports the benefits of Gliflozins for heart and kidney health, the number of prescriptions is going up, and they are being used more often in people who are at higher risk, such as those with heart failure and diabetes.

## Data Availability

The raw data supporting the conclusion of this article will be made available by the authors, without undue reservation.
